# γδ T cells recruitment and local proliferation in brain parenchyma benefit anti-neuroinflammation after cerebral microbleeds

**DOI:** 10.3389/fimmu.2023.1139601

**Published:** 2023-03-29

**Authors:** Xin Su, Shuxian Yang, Yanxiang Li, Zongqin Xiang, Qiao Tao, Shengde Liu, Zhinan Yin, Liyun Zhong, Xiaoxu Lv, Libing Zhou

**Affiliations:** ^1^ Guangdong Provincial Key Laboratory of Nanophotonic Functional Materials and Devices, South China Normal University, Guangzhou, Guangdong, China; ^2^ Guangdong-Hongkong-Macau Central Nervous System Regeneration (CNS) Institute of Jinan University, Key Laboratory of Central Nervous System Regeneration (CNS) (Jinan University)-Ministry of Education, Guangzhou, Guangdong, China; ^3^ Guangdong Provincial Key Laboratory of Brain Connectome and Behavior, Shenzhen Institute of Advanced Technology, Chinese Academy of Sciences, Shenzhen-Hong Kong Institute of Brain Science-Shenzhen Fundamental Research Institutions, Shenzhen, China; ^4^ CAS Key Laboratory of Brain Connectome and Manipulation, Shenzhen Institute of Advanced Technology, Chinese Academy of Sciences, Shenzhen-Hong Kong Institute of Brain Science-Shenzhen Fundamental Research Institutions, Shenzhen, China; ^5^ The Biomedical Translational Research Institute, Jinan University, Guangzhou, Guangdong, China; ^6^ Laboratory for Neuroscience in Health and Disease, Guangzhou First People’s Hospital School of Medicine, South China University of Technology, Guangzhou, China; ^7^ Guangdong Provincial Key Laboratory of Photonics Information Technology, Guangdong University of Technology, Guangzhou, Guangdong, China; ^8^ Guangdong Provincial Key Laboratory of Tumor Interventional Diagnosis and Treatment, Zhuhai Institute of Translational Medicine, Zhuhai People’s Hospital Affiliated with Jinan University, Jinan University, Zhuhai, Guangdong, China; ^9^ Department of Neurology and Stroke Center, The First Affiliated Hospital & Clinical Neuroscience Institute of Jinan University, Guangzhou, Guangdong, China; ^10^ Co-innovation Center of Neuroregeneration, Nantong University, Nantong, Jiangsu, China; ^11^ Neuroscience and Neurorehabilitation Institute, University of Health and Rehabilitation Sciences, Qingdao, Shandong, China; ^12^ Center for Exercise and Brain Science, School of Psychology, Shanghai University of Sport, Shanghai, China

**Keywords:** gamma-delta T cells, cerebral microbleeds, neuroinflammation, neuroprotection, live imaging

## Abstract

**Background:**

Cerebral microbleeds (CMBs) are an early sign of many neurological disorders and accompanied by local neuroinflammation and brain damage. As important regulators of immune response and neuroinflammation, the biological behavior and role of γδ T cells after CMBs remain largely unknown.

**Methods:**

We made a spot injury of microvessel in the somatosensory cortex to mimic the model of CMBs by two-photon laser and *in vivo* tracked dynamical behaviors of γδ T cells induced by CMBs using *TCR-δ^GFP^* transgenic mice. Biological features of γδ T cells in the peri-CMBs parenchyma were decoded by flow cytometry and Raman spectra. In wildtype and γδ T cell-deficient mice, neuroinflammation and neurite degeneration in the peri-CMBs cortex were studied by RNAseq, immunostaining and *in vivo* imaging respectively.

**Results:**

After CMBs, γδ T cells in the dural vessels were tracked to cross the meningeal structure and invade the brain parenchyma in a few days, where the division process of γδ T cells were captured. Parenchymal γδ T cells were highly expressed by CXCR6 and CCR6, similar to meningeal γδ T cells, positive for IL-17A and Ki67 (more than 98%), and they contained abundant substances for energy metabolism and nucleic acid synthesis. In γδ T cell-deficient mice, cortical samples showed the upregulation of neuroinflammatory signaling pathways, enhanced glial response and M1 microglial polarization, and earlier neuronal degeneration in the peri-CMBs brain parenchyma compared with wildtype mice.

**Conclusion:**

CMBs induce the accumulation and local proliferation of γδ T cells in the brain parenchyma, and γδ T cells exert anti-neuroinflammatory and neuroprotective effects at the early stage of CMBs.

## Introduction

Cerebral microbleeds (CMBs) are a significant and independent risk factor of intracerebral hemorrhage and considered as the early sign of many neurological disorders such as cognitive degeneration and stroke (1, 2). The size of CMBs varies from 5 to 240 µm in diameter. Focal hemosiderin deposition is the important pathological feature of CMBs, which is caused by the microvessel (less than 10 mm in diameter) leakage and readily recognized by magnetic resonance imaging (MRI) with very low signal intensity. In the brain, CMBs activate local neuroinflammation and oxidate stress response to damage neural tissue and function (3). There are many capillaries in the cortex, and CMBs may lead to blood clotting in the vascular lumen which subsequently affects local blood supply and causes tissue damage (4).

Studies of CMBs are mostly focused on endogenous brain cellular responses induced by active microglia and astrocytes (4, 5). Recent studies revealed that immune cells located in the dura meninges could play an important role in neurological diseases (6). The meninges are composed of dura, arachnoid, and pia structure, serve as a protective barrier for the central nervous system (CNS) as well as the bridge between the immune and the nervous system. Immune cells such as B cells, T cells, macrophages and other monocytes reside in the meninges, and are known to function in immune surveillance or mediate postnatal immune response (7). These cells may invade the brain parenchyma through the cerebrospinal fluid circulation and play a role in immune monitoring or removing harmful substances and metabolic waste from the brain in neurological diseases. In the experimental autoimmune encephalomyelitis (EAE) mouse model, autoreactive effector T cells enter the brain parenchyma by infiltrating the leptomeninges *via* the pial vasculature (7, 8). In severe brain hemorrhaging disease like hemorrhagic stroke, regulatory T cells are recruited from choroid plexus and express interleukin 10 (IL-10) and osteopontin to alleviate microglia activation at the acute injury stage (9, 10), and more immune cells including CD4^+^/CD8^+^ T cells, cytotoxic T lymphocytes and γδ T cells are gathered into the edema area by expressing massive inflammatory factors such as interleukin 21 (IL-21), interleukin 17 (IL-17), tumor necrosis factor α (TNF-α) or interferon-gamma (IFN-γ) to cause neuronal autophagy and apoptosis, inflammatory cascade, as well as proinflammatory differentiation of microphages at the later stage (11). On the other hand, CMBs cause minor bleeding and relative mild inflammation, but both resident glia cells and immune cells originating from the invasion of blood-borne monocytes are involved. Diverse immune responses occur within and around the lesion. Activation and proliferation of microglia and macrophages form an inflammatory microenvironment (12). It is reported that early penetration of antiviral CD8^+^ T cells in the mouse brain after a neurotropic virus infection alleviates CMBs induced by virus infection (13).

γδ T cells contain a small subset of T cells and participate in an immediate immune response by direct antigen recognition (14). In neurotrauma, γδ T cells are one group of the earliest immune cells arriving at the injury site (15–17). Moreover, γδ T cells in the meninges regulate the inflammatory responses during massive CNS injury by secreting typical immune factors: IFN-γ and IL-17 (18–20). Therefore, we assume that γδ T cells may be involved in the immune response and influence the pathological process in the brain after CMBs.

To test this hypothesis, we made a microvessel spot injury in the somatosensory cortex to mimic the model of CMBs by two-photon laser (21). Using *TCR-δ^GFP/+^* mice, we traced the moving trajectory and biological changes of γδ T cells after injury *in vivo* using a two-photon microscopy. Characteristics of γδ T cells in the brain parenchyma were studied by flow cytometry and Raman spectra analysis. The CMBs-induced neuroinflammation and brain damage were investigated by immunofluorescent staining and *in vivo* imaging of neuron morphology in control and γδ T cell-deficient (TCR-δ−/−) mice. RNA sequencing (RNAseq) of cortical samples disclosed the effect of γδ T cells deficiency on gene expression and signaling pathways. Our study identified the origin and biological characteristics of γδ T cells in the brain parenchyma after CMBs and demonstrated a beneficial role of γδ T cells at the early stage of CMBs.

## Materials and methods

### Animals

All animal experiments were approved by and performed in accordance with The Jinan University’s Institutional Laboratory Animal Care and Use Committee. All mice were C57BL/6 and the wildtype (WT) mice were purchased from Vital River Laboratory Animal Technology Co. Ltd, Beijing, China. The mice of *Thy1-YFP*, *TCR-δ* knockout (*TCR-δ^-/-^*), *Tcrd-H2B-EGFP* (*TCR-δ^EGFP^*), *TCR-α* knockout (*TCR-α^-/-^*) and *IFN-γ^EGFP^* were purchased from The Jackson Laboratory. The use of F1 heterozygotes from *TCR-δ^EGFP^* and *TCR-α^-/-^* mice were to genetically exclude highly fluorescent false-positive cells and named as *TCR-δ^EGFP/+^* in the later section. All mice were 7–8 weeks old and weighed 17–22 g at the time of surgery. Animals were maintained under pathogen-free conditions, with a 12-h day/night cycle.

### Reagents

APC anti-mouse IFN-γ (clone XMG1.2), PE-Cy7 anti-mouse CD3 (clone 145-2C11), BV421 anti-mouse TCR γ/δ (clone B1), PE anti- mouse CCR6 (clone 29-2L17), and PE anti-mouse Ki67 (clone 16A8) were purchased from BioLegend (San Diego, CA). 7AAD Viability Staining Solution were purchased from Tianjin Sungene (Tianjin, China). APC-Cy7 anti-mouse CD45 (clone 30-F11) and eFluor™ 506 anti-mouse IL-17A (clone eBio17B7) were from eBioscience. Hamster anti-mouse CD28 mAb (clone PV1) was purchased from Tianjin Sungene (Tianjin, China), rmIL-2 (CK24) from Novoprotein (Zhejiang, China), and hamster anti-mouse TCR γδ (clone UC7-13D5) purchased from BioXcell (West Lebanon, USA) were used for γδ T cell culture. The standard culture medium was RPMI 1640 (Euroclone, Milano, Italy), containing 10% fetal bovine serum (FBS) (Euroclone), 2 mM L-glutamine, 100 U/ml penicillin, and 100 mg/ml streptomycin. Fixation solution consisted of 0.1M phosphate-buffered saline solution (PBS) and 4% paraformaldehyde (PFA). Tissue digestion medium was High glucose Dulbecco’s Modified Eagle Medium (DMEM, Gibco), containing 4U/ml Collagenase type VIII (Sigma-Aldrich) and 50U/ml DNase I (Thermo Scientific) for the meninges, or 1U/ml Collagenase type IV (Sigma-Aldrich) and 50U/ml DNase I (Thermo Scientific) for brain tissue. BD IMag™ streptavidin particles plus - DM (BD Biosciences, USA) and biotin hamster anti-mouse γδ T-cell receptor antibody (clone GL3) were both from BD biosciences.

### Preparations for brain surgery

After anesthetization, mice were subjected to intraperitoneal injection of 3% dextran rhodamine B to label blood plasma. The head of the mouse was fixed using a head holder (Narishige STS-A SG-4N). An incision of the skin/scalp approximately 1 cm in length was cut using a surgical scalpel to expose the skull in the region of occipital lobes. Thinned-skull windows were generated using a high-speed micro drill, with a diameter of 200 μm. Further grinding of the skull, using a fine microsurgical blade, brought the bone to its thinnest area. The skull was removed, and a chronic glass-covered cranial window was implanted over the cortex. Vascular topography and the blood clot formed in CMBs were used as a guide for selecting the same region in the subsequent imaging sessions.

### The model of CMBs

The model was established as previously described (21). Briefly, mice were fixed on a payload platform, and femtosecond lasers were used to target a transverse capillary vessel in the cortex (80-100 μm deep from the vessel in the subarachnoid space, 5 μm in diameter), comparable to the size of the smallest bleeds in cerebral small vessel disease, rupturing the targeted vessel with short bursts of tightly focused femtosecond laser pulses with sufficient energy at the laser focus. The laser parameter was as follows: wavelength, 800 nm; frequency, 80 MHz; and pulse width, 140 (+/= 20) fs. A previous study disputed that naturally occurring microhemorrhages that caused blood clots may sometimes also occlude the vessel lumen, expecting more severe tissue damage from the resulting decrease in local blood flow (21). Therefore, we framed the radiation range slightly wider than the vessel wall. According to the output power, path loss, pass rate of the lens and software, the laser energy reaching the sample is about 20 J. Then CMB was produced in the cortex. Laser irradiation deposited relatively little total energy, so there was minimal collateral damage to the surrounding tissue from the laser itself. The ruptured wall of the targeted vessel clotted within a few seconds and restricted the size of the hematoma formed.

### Single cell suspension isolated from the brain and meninges

After anaesthetization, mice were perfused by ice-cold PBS using a 20 ml syringe with a 30 G needle until exsanguinated. The heads of the mice were severed just above the shoulder. Skin and muscle were removed with fine-angled scissors and a cut from the nasal bone was made to remove the skull. After the post-tympanic hook was removed, the lower portion of the skull was carefully cut clockwise around the skull with curved scissors and discarded. Then the brain was removed out of the upper half of the skull, and the skullcap was placed in a 24-well plate with 1 ml of ice cold DMEM. The meninges were completely removed from the skull with the tip of a very fine forceps and placed in the digestive medium (DMEM with 4 U/ml collagenase VIII and 50 U/ml DNase I) in a 37°C-water bath for 15 minutes. Filtrated by a 70 μm nylon mesh cell strainer, the meningeal cell suspensions were then centrifuged at 300×g at 4°C for 10 min and resuspended in cold 0.1 M PBS (pH 7.4) for all following experiments. Meanwhile, brain tissue from the injured cortex was removed and placed in ice-cold digestive medium (DMEM with 1U/ml collagenase IV and 50 U/ml DNase I) and shaken at a rate of 300 rpm, at 37°C for 30 min, with the intact contralateral cortex as a control. Fast pipetting was done to dissociate tissue, and the brain cell suspension was centrifuged at 300×g at 4°C for 10 min and resuspended with 40% Percoll (GE Healthcare Bio‐Science AB, Uppsala, Sweden). After centrifugation at 300×g at 20°C for 15 min, immune cell clots were precipitated on the bottom of the centrifuge tube.

### Flow cytometry analysis

Meningeal and brain single cells were suspended in a 24-well culture plate with 1 ml of DMEM culture medium containing 10% of FBS and treated with phorbol myristate acetate (PMA; 10 ng/ml), Golgi Plug (1:1000) and ionomycin (1 μg/ml) for 5 h (37°C, 5% CO_2_), then permeabilized with 0.5% (w/v) saponin. After washing with PBS, cells were labeled with cell surface staining marker at a 1:100 dilution: FITC-conjugated anti-mouse CD3, BV421 anti-mouse TCR-γδ, PE-Cy7 anti-mouse CXCR6, PE anti-mouse CCR6, and APC-Cy7 anti-mouse CD45 for 20 min at 4°C and 7AAD was added to label live cells, following detection by BD FACS Verse flow cytometry. As for intracellular and intranuclear staining, cells were intracellularly stained with APC-anti mouse IFN-γ, BV510 anti-mouse IL-17A, and PE anti-mouse Ki67 using the protocol described in Foxp3/Transcription Factor Staining Buffer Set (Thermo Fisher, 00-5523-00) staining for 30 min at 4°C and washed with PBS. Data were analyzed using FlowJo (TreeStar).

### Immunofluorescent staining

For meninges lymphatic staining, skulls were trimmed into round and flat shaped parietal bones with ophthalmic scissors, then incubated in blocking solution (0.3% PBST with 5% BSA and 3% donkey serum) for 2 h followed with primary antibody mouse anti-mouse Lyve-1 (1: 200, clone ALY7, eBioscience) for 18 h at 4°C and washed with PBS thrice before staining with secondary antibody donkey anti-mouse Alexa Fluor™ Plus 594 (1:500, A32744, Invitrogen). Immunofluorescent staining of brain slices were prepared and processed as previously described (15). Briefly, mouse brains were fixed and obtained followed by cardiac perfusion with PBS and 4% PFA, and then dehydrated with gradient sucrose before being cut into 35 mm slices in the coronal plane around the injured site with a sliding microtome. Brain slices were subjected to routine immunofluorescent staining. Primary antibodies included: rabbit anti-mouse Iba1 (1:500, 019-19741, Wako), rabbit anti-mouse glial fibrillary acidic protein (GFAP; 1:500, ab7260, Abcam), rat anti-mouse CD11b (1:500, ab8878, Abcam), rabbit anti-mouse Arginase1 (Arg1; 1:300, ab91279, Abcam) or mouse anti-mouse inducible nitric oxide synthase (iNOS; 1:300, EPR16635, Abcam). The signal was disclosed by secondary antibodies: donkey anti-rabbit IgG (H+L) Alexa Fluor™ Plus 594 (1:500; A-21207, Thermo Fisher Scientific) or donkey anti-mouse IgG (H+L) Alexa Fluor™ Plus 546 (1: 500; A10036, Thermo Fisher Scientific), and donkey anti-rat IgG (H+L) Alexa Fluor™ Plus 488 (1:500; A-21208, Thermo Fisher Scientific). The meninges were soaked in PBS and imaged with a two-photon microscope (LSM780, Zeiss, Germany). Slices were sealed with fluorescent mounting medium containing DAPI (62248, Thermo Scientific) and imaged with a fluorescent microscope (Imager Z2, Zeiss, Germany).

### γδ T cell culture

For expansion of γδ T cells from mice *in vivo*, splenic γδ T cells were sorted and expanded with anti-γδ antibodies. Briefly, γδ T cells were polarized by plate-coated anti-CD3 (10 mg/mL), soluble anti-CD28 (1 mg/mL), and IL-2 (2 ng/mL) in 1640 medium. After 6 days of proliferation of γδ T cells, positive selection was performed with the BD IMag™ Cell Separation Magnet.

### Raman spectra analysis

A Renishaw in *Via* Raman spectrometer attached to a Leica upright microscope equipped with a 100× objective, a 633 nm laser with a laser power of 50 mw, a laser spot size of 1 μm, and a CCD detector with a spectral resolution of 0.99 cm was utilized to collect the Raman spectra of individual cells for all cell types, in which five different areas were randomly probed within a cell and the integration time for acquiring a Raman spectrum of a cell was 10 s in all experiments. A Raman spectrum of each cell type was expressed by an average of 10 cells in the scan range of 400 to 1800 cm. Raman spectroscopic data processing was performed with MATLAB and Origin software. In this study, γδ T cells were sorted by fluorescence under the microscope to measure Raman spectrum on the fifth day after CMB. Naive γδ T cells were also separated from the spleen as the control group and sorted by the same method. Each spectrum is the average of more than 10 cells. All spectra were normalized to the area to perform a direct comparison of the data. Background subtraction was also done.

### Neuron tracing

To sparsely label cortical neurons, AAV9-CMV-GFP (1E+13 μg/ml, 500 nl/injection; Vigene Biosciences Branch) was injected into bilateral the motor cortex ( ± 1.5 mm lateral to the midline, 2 mm anteroposterior to the bregma, 0.2 mm in depth) respectively. The model of CMBs and *in vivo* imaging of labelled neurons were proceeded 3 weeks later.

### Statistical analysis

The SPSS 19.0 statistical software was used to process and analyze the data and images. Data were presented as mean ± SEM. The comparisons were performed using Student’s *t*-test or One-way ANOVA with Tukey’s multiple comparison. The level of significance was set at 0.05.

## Results

### γδ T cells are recruited from dural meningeal vessels to brain parenchyma after CMBs

**Figure 1:**
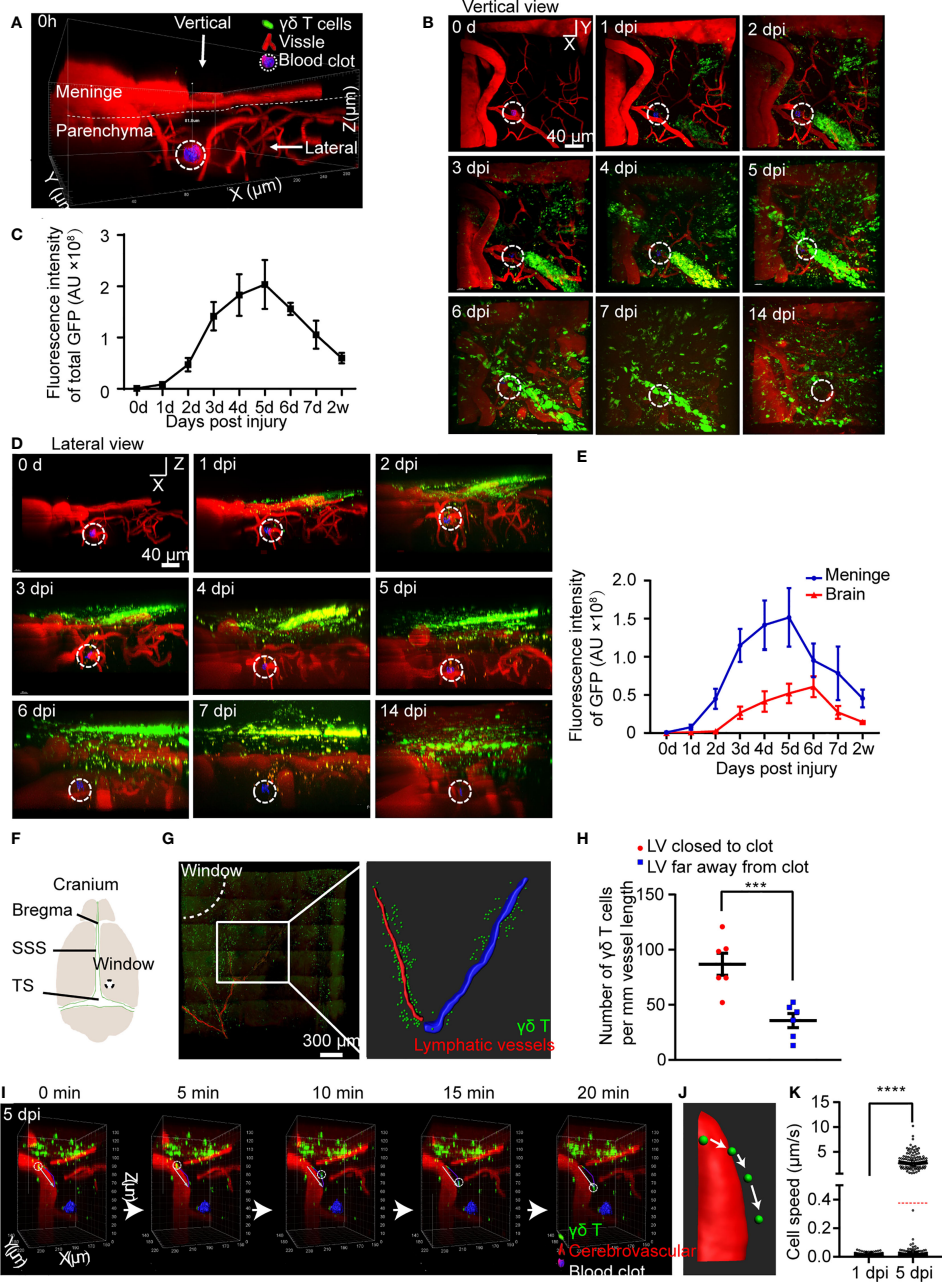
CMBs induce migration of meningeal γδ T cells to brain parenchyma. **(A–E)** *In vivo* imaging of γδ T cells (green) and blood vessels (red) after CMBs (dotted circles) using *TCR-δ ^EGFP/+^* mice. Reconstruction of stacked sections shows the distribution of γδ T cells at different days post injury (dpi) through the vertical **(B)** and lateral **(D)** view. Total γδ T cells disclosed by GFP intensity gradually increase from 1 to 5 dpi and then decrease **(C)**; mean ± SEM, n≥3 mice). **(F)** Schema illustrates opening window (dotted circle) in the cranium (SSS: superior sagittal sinus, TS: transverse sinus). **(G, H)** Relative location of meningeal lymphatic vessels (LVs; anti-Lyve-1 immunostaining, red in left panel) and γδ T cells (green) shows that the LV closer to the injury spot (the LV indicated by drawing red line, right panel) is surrounded by more γδ T cells than that farther away from the injury site (the LV indicated by drawing blue line, right panel) **(G)**, with a significant increase indicated by quantification of γδ T cells **(H)**; n=3 mice, Student’s *t-test*, *** *P*<0.001). **(I–K)** *In vivo* imaging reveals the dynamic movement of γδ T cells in the brain parenchyma **(I)**; reconstructed images indicate the moving trajectory **(J)**, and statistical analysis shows a significant increase of moving speed at 5 dpi compared with that at 1 dpi **(K)**; average speed indicated as the red dotted line; **** *P*<0.0001, Student’s *t*-test, n ≥ 8 mice).

To study the response of γδ T cells to CMBs, γδ T cells were dynamically tracked using *TCR-δ^EGFP/+^* transgenic mice in which γδ T cells were labelled by EGFP, and the blood vessels were visualized by intraperitoneal injection of rhodamine before injury. After opening a cranial window, an injury (around 25 μm^2^ area) on the capillary vessel (about 5 μm in diameter) at the brain parenchymal surface of the somatosensory cortex was made by a high energy two-photon laser to mimic a model of CMBs. *In vivo* long-term imaging was performed using two-photon microscopy and the relative position of blood vessels was used as reference (**Figure 1A**). We observed a small number of γδ T cells gathering in the dural site close to the injury spot at 6 h and 1 day post injury (dpi), indicating they response rapidly to hemorrhage (**Figure 1B**, **Supplementary Figure 1A**). The number of aggregated γδ T cells increased significantly at 3 dpi, when a tubular distribution was gradually formed in the dura (**Figure 1B**, **Supplementary Figure 1A**). To verify the permeability changes of meningeal vessels, Evans blue was injected through caudal vein, showing a large amount of dye leakage to the cortex of brain parenchyma in animals with CMBs but rare in sham animals with opening cranial window only (**Supplementary Figure 1B**). This indicates that CMBs, but not cranial window surgery, induces the increase of blood vessel permeability.

After CMBs, total fluorescent intensity of γδ T cells gradually increased from 1 dpi, peaked at 5 dpi, and decreased afterwards (**Figures 1B, C**). The distribution of γδ T cells was readily identified in the brain parenchyma and the meninges from the lateral view (**Figure 1D**, **Supplementary Figure 1A**). Their number in the brain parenchyma peaked at 6 dpi, while peaked 1 day earlier in the meninges (**Figures 1D, E**). Many γδ T cells were still detectable in the meninges after 2 weeks of CMBs (**Figures 1B–E**), indicating a long-term residence of meningeal γδ T cells after CMBs. In contrast, no γδ T cells were observed in sham animals with opening cranial window only from 1 dpi to 5 dpi (**Supplementary Figure 1C**). It was reported that basal meningeal lymphatic vessels were the major routes for cerebrospinal fluid macromolecular uptake and drainage (22), and antigens from the brain parenchyma were preferentially absorbed by meningeal lymphatic vessels to activate the immune cells around and induce the response of the cervical lymphatic immune system (23). To test whether γδ T cells were also recruited through lymphatic vessels, we stained the whole meninges at 5 dpi using anti-Lyve-1 and conducted a 3D reconstruction of the lymphatic vessels (LVs). We found that most γδ T cells accumulated around the capillary branches of meningeal LVs close to the injury spot and much less surrounded the same level branches relatively farther to the injury spot (**Figures 1F–H**). However, no γδ T cells were observed inside the lumen of the LVs (**Supplementary Figure 1D**).

To study the dynamic movement of γδ T cells after CMBs, we carried out a 20-minutes continuous imaging using a two-photon microscopy. γδ T cells detached from the wall of blood vessels, rolled toward the injured site, and then penetrated down into the brain parenchyma (**Figures 1I, J**; **Supplementary Video 1**). Following heterogeneous trajectories, the average moving speed of γδ T cells was gradually increased, which was 0.0077 ± 0.0064 μm/s at 1 dpi (964 cells from 3 animals) and 0.38 ± 1.12 μm/s at 5 dpi (1219 cells from 3 animals) after CMBs, showing a significant increase at 5 dpi (**Figure 1K**, **Supplementary Figure 1E**, *P*<0.0001, n=3). The moving speed of γδ T cells changed in different locations and over time. At 1 dpi, the maximal moving speed of γδ T cells was 0.05 μm/s, but the percentages of γδ T cells with more than half of the maximal speed (0.025 μm/s) were not the same in different regions: 40.0 ± 3.7% in the parenchyma, 24.0 ± 2.2% in the subarachnoid space, and 17.3 ± 1.3% in the dura. Higher percentage of γδ T cells with the moving speed more than 5 μm/s was observed in the subarachnoid space compared with those in the parenchyma and dura at 5 dpi (**Figure 1K**).

Aforementioned results demonstrate that CMBs induce the permeability increase of the dural blood vessels, and the active γδ T cells are attracted from the dural blood vessels to invade the peri-CMBs brain parenchyma. To further confirm this, we cultured γδ T cells from *TCR-δ^EGFP/+^* mice and transplanted them to *TCR-δ^-/-^* mice *via* tail vein delivery. After CMBs, the numbers of γδ T cells were gradually increased in the brain parenchyma surrounding the injury site and reached the peak at 4 dpi (**Supplementary Figures 2A, B**).

### γδ T cells in the brain parenchyma proliferate locally and secrete IL-17A

**Figure 2:**
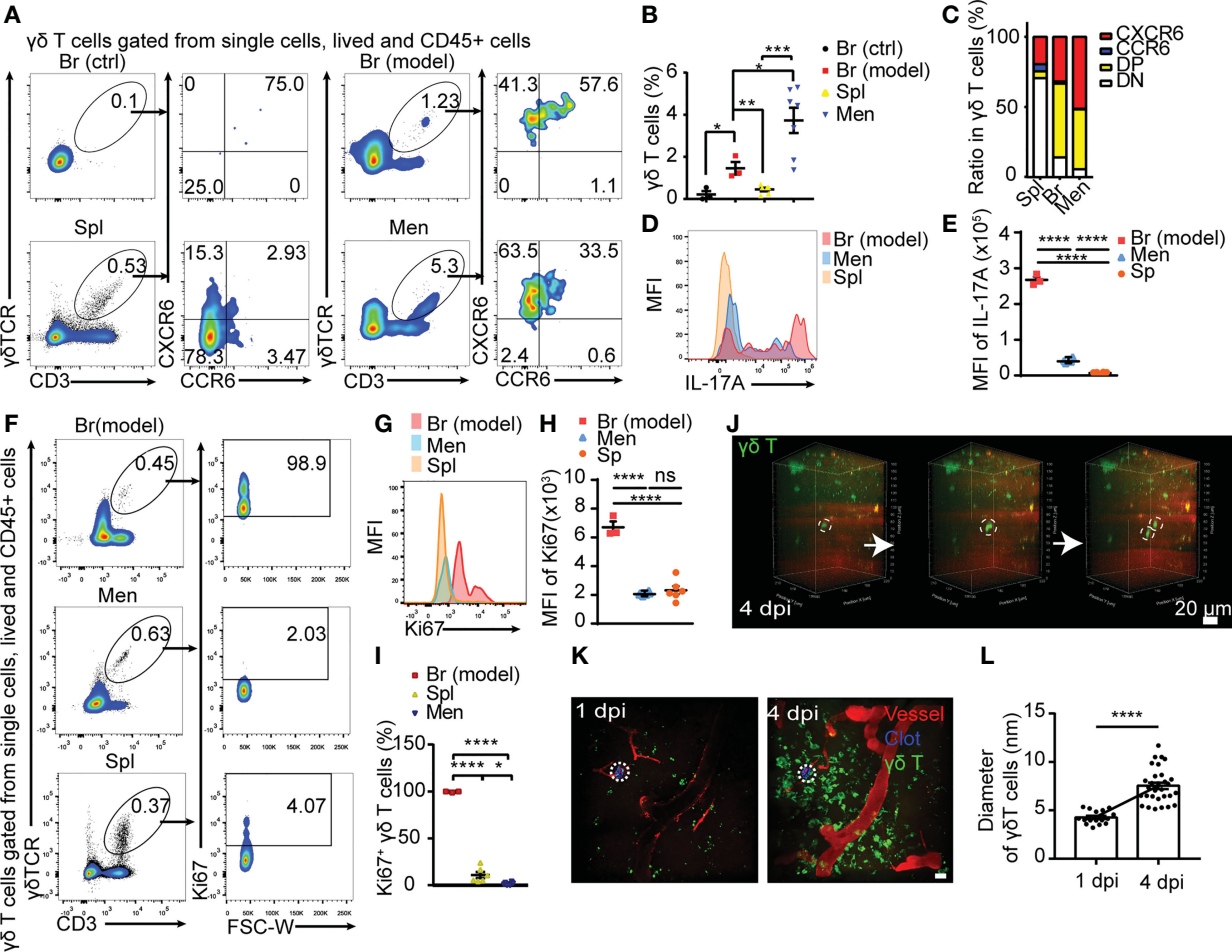
Meningeal γδ T cells migrating to peri-CMBs brain parenchyma are activated to proliferate and secret IL-17A. **(A)** The properties of γδ T cells sorted from peri-CMBs parenchyma (Br-model), contralateral cortex (Br-ctrl), meninges and spleen (Spl) of WT mice at 5 dpi are identified by flow cytometry. **(B, C)** Statistical analysis of flow cytometry shows that γδ T cells are significantly increased in peri-CMBs parenchyma **(B)**; one sample from 2-3 mice and n≥3 samples in each group) and γδ T cells of peri-CMBs parenchyma and meninges are characterized by the elevated expression of CXCR6 and CCR6 **(C)**; DP: CCR6 and CXCR6 double positive; DN: double negative; * *P*<0.05; ** *P*<0.01; *** *P*<0.001; n=7, One-way ANOVA with Tukey’s multiple comparison). **(D, E)** High expression of IL-17A is identified in γδ T cells of peri-CMBs parenchyma by measuring mean fluorescence intensity (MFI) at 5 dpi (3 independent experiments in each group). **(F–I)** High expression of Ki67 is identified in parenchymal γδ T cells at 5 dpi using flow cytometry. The percentages of Ki67-positive γδ T cells derived from different tissues **(F)**, mean fluorescence intensity of Ki67 expression **(G, H)** and the statistical analysis of Ki67-positive γδ T cells from peri-CMBs parenchyma, meninges and spleen **(I)**; one sample from at least 3 mice and n≥3 samples in each group; **** *P*<0.0001; One-way ANOVA with Tukey’s multiple comparison). **(J)** Time-series snapshots at 5-min intervals shows the division of γδ T cells in brain parenchyma at 4 dpi. **(K, L)** The diameters of γδ T cells (green) in peri-CMBs parenchyma are significantly increased at 4 dpi compared with them at 1 dpi (blood vessels labelled by rhodamine in red; dot circles: injured sites, Student’s *t*-test).

To further investigate the biological characteristics of γδ T cells, we collected brain parenchyma and meninges from the region surrounding the CMBs, brain parenchyma from the intact side, and spleen at 5 dpi. Tissues were lysed into single cells and isolated T cells were subjected to intra/extra cellular and live/dead staining for flow cytometry. The percentage of γδ T cells in CD45^+^ lymphocytes was less than 0.1% in the intact cortex and around 0.6% in the spleen, whereas it was significantly increased in the injured brain (more than 1%) and the meninges (more than 4%) (**Figures 2A, B**). γδ T cells from mouse meninges at 5 dpi were characterized by the expression of CXCR6 and CCR6 (**Figures 2A–C**), which was consistent with the previous report (24). High expression of CXCR6 and CCR6 (single or double positive) was also found in γδ T cells from the injured brain parenchyma, but not in the spleen or intact-side brain parenchyma (**Figures 2A–C**), indicating that γδ T cells of the injured brain parenchyma and meninges are homologous.

IFN-γ and IL-17A are two main effectors of γδ T cells and involve in the regulation of neuroinflammation in CNS diseases (15, 18). The ratio of IL-17A^+^ γδ T cells was dramatically increased in the meninges and parenchyma compared with those from spleen (**Figures 2D, E**). Higher proportion of IL−17A^+^ γδ T cells was found in the peri-CMBs parenchyma compared with IFN-γ^+^ γδ T cells after the stimulation of PMA and ionomycin (**Supplementary Figures 3A–D**), while the expression of IL-17A was extremely low in cells without stimulation (**Supplementary Figures 3E–G**), proving γδ T cells were further stimulated and activated into γδ T17 in the parenchyma.

Ki67 is a typical marker of cell proliferation. Here we used Ki67 to present the proliferation rate of γδ T cells from spleen, meninges and brain parenchyma with flow cytometry. The percentage of Ki67^+^ γδ T cells was 98.9% in the injured brain tissue, 2.03% in meninges and 4.07% in the spleen (**Figures 2F, I**) and the mean fluorescence intensity of Ki67 expression showed the similar change (**Figures 2G, H**), suggesting that γδ T cells in the injured site are in exuberantly proliferative state. In live animals with *in vivo* imaging, we also captured the process of γδ T cells dividing and morphological change in the injured parenchyma by two-photon microscopy at 1 dpi or 4 dpi (**Figures 2J, K**; **Supplementary Videos 2**, **3**). The diameters of γδ T cells were significantly increased at 4 dpi compared with those at 1 dpi (*P*<0.0001, **Figure 2L**), indicating a higher activation state at the later stage of CMBs.

### Raman spectra shows CMBs-recruited γδ T cells in an active state of transcription and lipid metabolism

**Figure 3:**
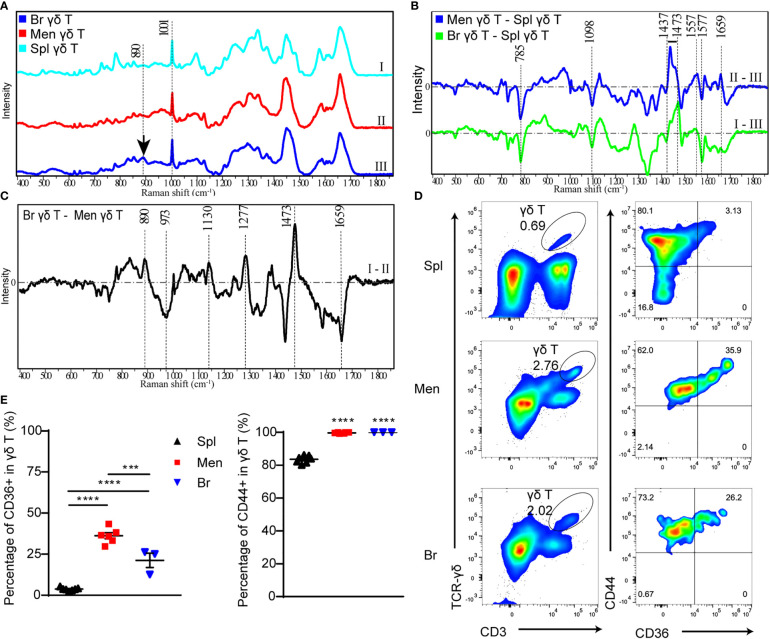
The components and functional state of γδ T cells are analyzed by Raman spectra and flow cytometry respectively. **(A)** Raman spectra of γδ T cells isolated from spleen (I), peri-CMBs brain parenchyma (II) and meninges (III) at 5 dpi. **(B, C)** The differential Raman spectrum of γδ T cells in meninges (Men) and spleen (Spl), brain parenchyma (Br) and spleen, brain and meninges. **(C)** Raman spectra showing characteristic peaks of saturated fatty acids (890cm^-1^, 1130cm^-1^, 1277cm^-1^) in peri-CMBs cortical tissues at 5 dpi. **(D, E)** Flow cytometry uncovers the percentages of CD36- or CD44-positive γδ T cells from the meninges, peri-CMBs parenchyma and spleen **(D)**, and statistical analysis shows high expression of CD44 and CD36 in γδ T cells from the meninges and peri-CMBs parenchyma compared to those from the spleen **(E)**. γδ T cells are gated on singled, lived, and CD45^+^ lymph cells. Each sample from 2 to 4 mice; n≥3 samples in each group; *** *P*<0.001, **** *P*<0.0001; One-way ANOVA with Tukey’s multiple comparison.

The process of cell proliferation and migration requires material synthesis and energy supply, accompanied by changes in cell components. When the photons of the excitation light interact with the specific chemical bonds or groups contained in the molecules, the photon frequency changes can be detected by Raman spectra as characteristic peaks. According to this theory, Raman spectra is generally used to disclose cell components and functional states (25). We compared the major substances in sorted single γδ T cells from the meninges, injured brain parenchyma, and spleen at 5 dpi using Raman spectra. All samples from different tissues showed a typical spectra of 1001 cm^-1^ (**Figure 3A**), a characteristics of cells enriched in phenylalanine (26). Spectral subtraction between parenchymal or meningeal and splenic γδ T cells showed the decreased peak at 785 cm^-1^ and 1098 cm^-1^ (**Figure 3B**), representing a unwinding state of nucleic acids for active transcription (25, 27), and the increase of the spectra peak of 1437-1471 cm^-1^ (**Figure 3B**) reflected CH deformation of lipids/protein associated with transcriptive process (25, 27). These results indicate γδ T cells in the brain parenchyma and meninges are responsive and considerably activated after CMBs.

To further analyze the specific components of γδ T cells infiltrated in the brain parenchyma, we subtracted the spectra of γδ T cells in the meninges from the brain parenchyma. Differential spectrum showed that peaks at 890cm^-1^, 1277 cm^-1^ and 1130 cm^-1^, representing the levels of C-O-O skeleton, δ (a helical structure of =CH) and saturated fatty acid chain of triacylglycerols respectively (28), were significantly increased in brain parenchymal γδ T cells (**Figure 3C**). In addition, the peak at 1473 cm^-1^ representing ring DNA and RNA was also increased in brain parenchymal γδ T cells (**Figure 3C**), indicating the changing form of DNA or RNA molecules in γδ T cells during invasion.

Lipids are one of the most important constituents of brain tissue, and are extensively involved in intracellular signaling processes (29). Moreover, accumulated studies showed the phagocytic capacity of γδ T cells for particles and antigens (30–32). To meet the requirement of energy-demanding and formation of new cellular components such as membranes, DNA, and proteins for increased cell size and proliferation, T cells are heavily dependent on extra and intracellular fatty acid content for their function (33). We collected brain parenchymal samples in the region with microvessel lesion at 5 dpi for Raman spectra. The characteristic peaks of saturated fatty acids (890cm^-1^, 1277 cm^-1^ and 1130 cm^-1^) were remarkable (**Figure 3C**), indicating that these tissues contain enriched fatty acids to support the lipid metabolism or produced by endocytosis process of brain parenchymal γδ T cells during proliferation and migration.

To confirm the result in Raman spectra, we proceeded flow cytometry to analyze the meningeal, splenic and parenchymal γδ T cells using antibodies including anti-CD36 (clone HM36), a lipid transport receptor of T cells, and anti-CD44 (clone IM7) as an activation marker (34, 35). Higher expression of CD36 was identified in γδ T cells from the meninges and brain parenchyma compared to those from the spleen (**Figures 3D, E**). In addition, most meningeal and parenchymal γδ T cells (nearly one hundred percentage) were positive for CD44 (**Figures 3D, E**). The results indicate that γδ T cells in the meninges and brain parenchyma are activated by CMBs and act as the scavenger of lipids.

Taken together, the Raman spectrum analysis demonstrates that γδ T cells from lesioned brain parenchyma are enriched in the components for cell metabolism and nucleic acid synthesis, supporting their proliferation and migration, and γδ T cells from the lesioned brain parenchyma share the similar characteristics with cultured γδ T17 cells, the origin of IL-17A secretion.

### γδ T cell deficiency enhances local neuroinflammation after CMBs

**Figure 4:**
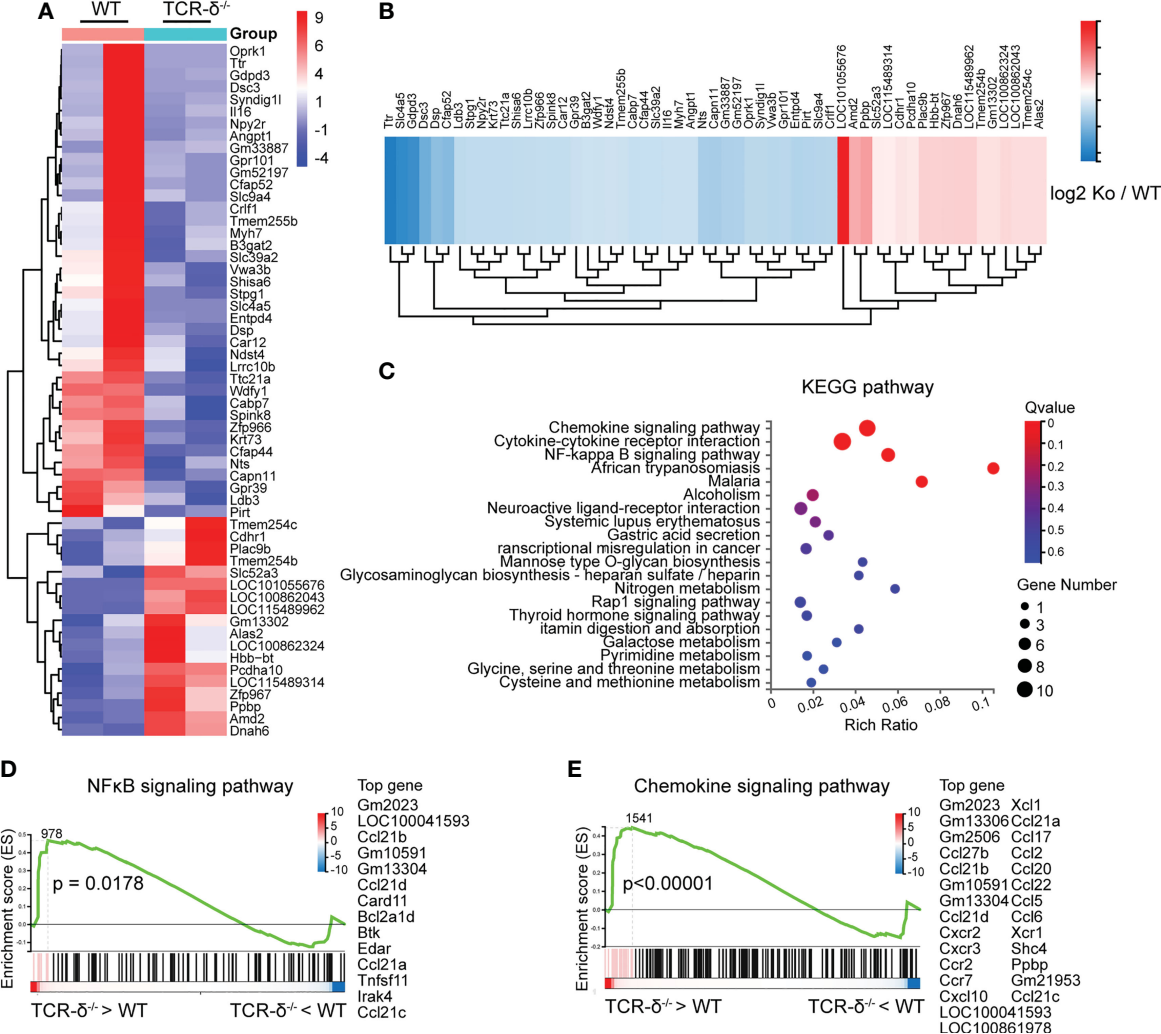
γδ T cells deficiency enhances inflammatory signaling pathways after CMBs. RNA-seq of peri-CMBs cortical samples from WT and *TCR-δ^-/-^* mice at 5 dpi. **(A)** Heatmap of gene expression in two groups. **(B)** Heatmap showing fold changes of differentially expressed genes between two groups. **(C)** KEGG pathway analysis of differentially expressed genes. **(D, E)** Gene set enrichment analysis (GSEA) showing the upregulation of NFκB and chemokine signaling pathways in *TCR-δ^-/-^* mice compared with WT mice (n=4 mice, 2 mice in one sample, *P* value of log2 (ko/wt) < 0.05).

Given that γδ T cells were highly activated in the parenchyma after CMBs, we further studied their role in the neuroinflammatory response by comparing the RNA-seq data of brain tissues from wildtype (WT) and γδ T cell deficient (*TCR-δ^-/-^*) mice at 5 dpi (**Figure 4A**). There were 57 differentially expressed genes (DEGs) between two groups, 18 DEGs with upregulation and 39 DEGs with downregulation in mutant samples (**Figures 4A, B**). KEEG pathway analysis showed that the most abundant DEGs were clustered into immune response signaling pathways such as chemokine signaling, cytokine-cytokine receptor interaction and NF-κB signaling (**Figure 4C**). The gene-set enrichment analysis showed the increase of NF-κB signaling and chemokine signaling in γδ T cell deficient samples compared with WT samples (**Figures 4D, E**).

**Figure 5:**
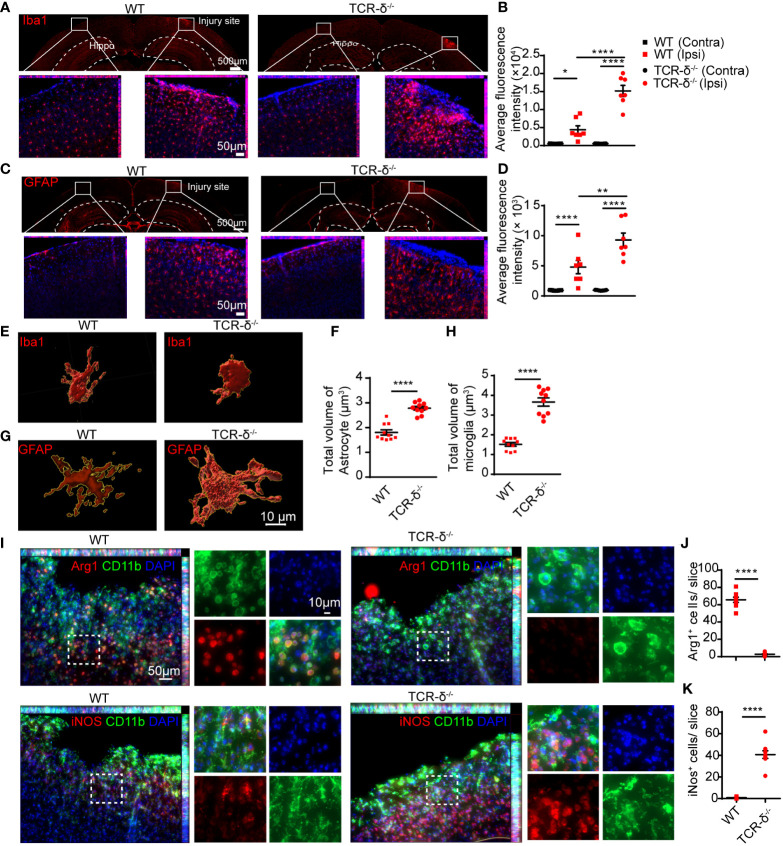
γδ T cells deficiency increases glial response and macrophage polarization in the cortex after CMBs. Immunofluorescent staining of cortical sections from WT and *TCR-δ^-/-^* mice at 5 dpi. **(A, B)** Anti-Iba1 immunostaining (red) shows increased active microglia surrounding the injury sites compared to the intact sides in both groups **(A)**, and the average fluorescence intensity are significantly higher in *TCR-δ^-/-^* mice compared to WT mice on the injury side **(B)**; * *P*<0.05, **** *P*<0.0001; One-way ANOVA with Tukey’s multiple comparison; n≥3 per group). **(C, D)** Similar increase of astrocyte response is identified in *TCR-δ^-/-^* mice in anti-GFAP immunostaining **(C)**, statistical analysis of average fluorescence intensity **(D)**; ** *P*<0.01, **** *P*<0.0001; One-way ANOVA with Tukey’s multiple comparison; n≥3 mice in each group). **(E–H)** Tridimensional reconstruction analysis show the significant increases of reactive microglia and astrocyte volumes in *TCR-δ^-/-^* mice compared to WT mice, respectively (**** *P*<0.0001; Student’s *t*-test). **(I–K)** Anti-CD11b double immunostaining with anti- Arg1 or anti- iNOS discloses M2 and M1 macrophages in the peri-CMBs regions respectively **(I)**. Statistical analysis of cell densities shows a decrease of M2 (anti-inflammatory) macrophages **(J)** and an increase of M1 (pro-inflammatory) macrophages **(K)** in *TCR-δ^-/-^* mice compared with WT mice (**** *P*<0.0001; Student’s *t*-test; n≥3 mice in each group; DAPI counterstained nuclei in blue; Hippo, Hippocampus; Ipsi, ipsilateral side; Contra, contralateral side).

To further investigate the role of γδ T cells involved in neuroinflammation after CMBs, we prepared brain sections for anti-Iba1 immunofluorescent staining from WT and *TCR-δ^-/-^* mice at 5 dpi. Increased active microglia were visible surrounding the lesion areas compared to the intact sides in both groups (**Figures 5A, B**). Statistic showed a significant increase of Iba1-immunoreactive density in the mutant compared to the WT on the injury side (**Figure 5B**). Similarly, more reactive astrocytes were found surrounding the lesion region in γδ T deficient animals after CMBs as shown in anti-GFAP immunostaining (**Figures 5C, D**). Tridimensional reconstruction of microglia and astrocytes in surrounding the lesions showed the significant increases of their volumes in *TCR-δ^-/-^* mice compared to WT mice (**Figures 5E–H**). Double immunofluorescent staining of CD11b (a marker for macrophage) with anti-iNOS (a marker for M1 microglia) or anti-Arg1 (a marker for M2 microglia) showed the significant increase of M1 macrophages and a significant decrease of M2 macrophages in the lesioned cortex from the *TCR-δ^-/-^* compared to the WT (**Figures 5I–K**).

Thus, γδ T cell deficiency induces an aggravated inflammatory reaction during the early stage of CMBs and aggregated γδ T cells in injured brain parenchyma contribute to anti-neuroinflammation.

### γδ T cell deficiency accelerates CMBs-induced neuron degeneration in the cortex

**Figure 6:**
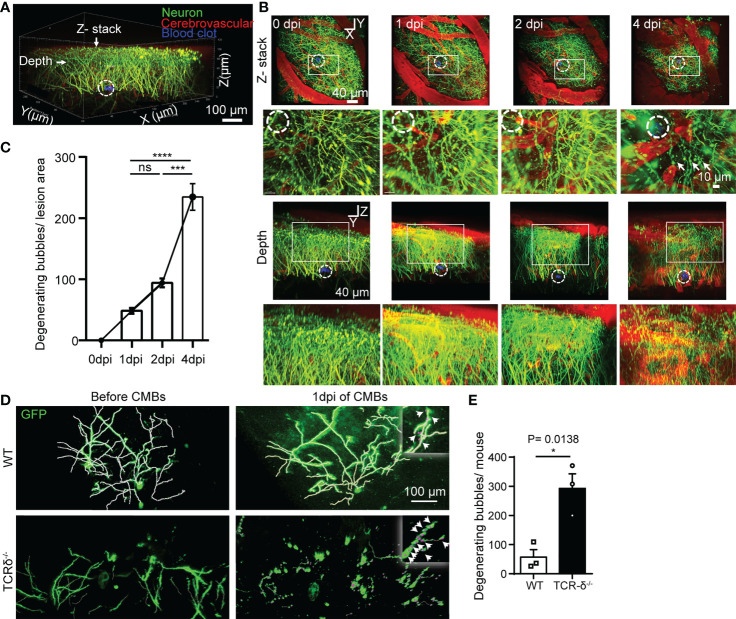
γδ T cells deficiency accelerates CMBs-induced neuron degeneration. **(A–C)** *In vivo* imaging of pyramidal neurons surrounding the injury site (dotted circles) at different timepoints after CMBs using *Thy1-YFP* mice. From lateral and vertical view, reconstruction of stacked sections **(B)** and statistical analysis **(C)** shows abundant degenerating neurite bubbles at 4 dpi, but very rare before 4 dpi (blood vessels labelled by rhodamine in red; enlarger images in B from rectangular frames upper respectively; *** *P*<0.001, **** *P*<0.00001; One-way ANOVA with Tukey’s multiple comparison; n=3 mice in each group). **(D, E)** Spare neurons in the cortex are labelled using AAV9-CMV-GFP virus in WT and *TCR-δ^-/-^* mice. Neurites are well defined with no degenerating bubbles before CMBs in both groups. Many degenerating neurite bubbles are visible in the *TCR-δ^-/-^* at 1 dpi of CMBs, but a few in the WT **(D)**; arrows in the insets). Statistics shows a significant increase of degenerating neurite bubbles in the mutant (**E**; * *P*<0.05; Student’s *t*-test; n=3 mice in each group).

CMBs are an early sign of brain damage. To identify the effect of CMBs on cortical neurons, we *in vivo* imaged neurites of pyramidal neurons surrounding the lesion using *Thy1-YFP* transgenic mice. Using a two-photon microscopy, scanned images showed that abundant GFP-labelled neurites were intermingled with cerebrovascular vessels traced by rhodamine (**Figure 6A**). At 4 dpi of CMBs, many YFP-positive degenerating neurite bubbles appeared surrounding and above the injury site, whereas rare of them were visualized at 0, 1 or 2 dpi (**Figures 6B, C**). The result indicates that CMBs induce a later, but not an immediate onset of neuron degeneration in the cortex.

To better visualize morphology of single neurons in the cortex, the virus of AAV9-CMV-GFP was injected into the cortex, and CMBs were made 3 weeks later using a two-photon microscopy. Scanned images readily showed neurites of sparsely labeled cortical neurons in WT and *TCR-δ^-/-^* mice before CMBs (**Figure 6D**). At 1 dpi of CMBs, rare degenerating neurite bubbles were identified surrounding the lesion in WT animals, but many of them were seen in *TCR-δ^-/-^* mice (**Figure 6D**). Statistic showed a significant increase of neuron degeneration in γδ T cell deficient mice (**Figure 6E**). Thus, γδ T cells deficiency enhances CMBs-induced neuron damage and recruited γδ T cells may play a neuroprotective role after CMBs.

## Discussion

In this study, we decoded the origin, biological properties, and roles of γδ T cells in the parenchyma at the early stage after CMBs. Our main findings include: (i) CMBs induce the accumulation of γδ T cells in the brain parenchyma from dural vessels by passing through the meningeal structures; (ii) γδ T cells are in the active state undergoing proliferation and movement locally in the brain parenchyma; (iii) γδ T cells mainly secrete IL-17A in the brain parenchyma; (iv) γδ T cells in the brain parenchyma have a beneficial role of anti-neuroinflammation and neuroprotection.

The meninges are a unique interface between the peripheral vasculature and the brain parenchyma, where peripheral γδ T cells are recruited and aggregated (36, 37). However, the function of meningeal γδ T cells in brain hemorrhage is blurry (38). The intact cortex is free of γδ T cells because the meningeal structures prevent their invasion into the brain parenchyma. Using a two-photon microscopy, we readily traced the movement of γδ T cells *in vivo* after CMBs. Based on the anatomical structure, γδ T cells subsequently pass through the dura, the arachnoid, the subarachnoid space, the pia, and finally invade the parenchyma surrounding the lesioned microvessel. This prediction is supported by the dynamic change of γδ T cell distribution: few at 0 dpi, some inside and outside of the dural vessels above the injury site at 1dpi, increasing cells scattered in the parenchyma and the meningeal structure thereafter. Imaging *in vivo* discloses the moving trajectory of single γδ T cells after CMBs, showing that their moving velocity is gradually increased. The peak of γδ T cell density in the parenchyma is one day later than that in the meninges, and this attributes to the time cost of γδ T cells movement from the dura to the parenchyma. Furthermore, flow cytometry shows that γδ T cells in the brain parenchyma share the same property of those in the meninges by the expression of CXCR6 and CCR6 (24) (**Figure 2A**). The CMBs in the brain parenchyma may activate the response of the dural microvessel to increase the permeability as shown in Evans blue staining and induce the recruitment of γδ T cells to the lesion, which should be due to the CCR6/CXCR6-mediated chemotactic effect. The relative location of γδ T cells and lymph vessels studied by double immunostaining indicates that parenchymal γδ T cells are not transported through the lymphatic vessels. Thus, our study demonstrates that the CMBs recruit γδ T cells from the dural blood vessels to the brain parenchyma. This is consistent with a previous report that dural blood vessels were the main route for immune cells to enter the meninges and cerebrospinal fluid in EAE models (37).

Peripheral macrophages may be recruited and proliferate in the peri-CMBs region to involve the brain response to a single bleed (12). Here we found similar features of γδ T cells. CMBs-recruited γδ T cells in the brain parenchyma undergo local proliferation, which biological behavior is supported by the following studies. Firstly, the diameters of parenchymal γδ T cells are significantly increased from 1 dpi to 4 dpi of CMBs, and the cell division is captured by *in vivo* imaging using two-photon microscopy. Secondly, the percentage of active γδ T cells is much higher in the lesioned parenchyma than those from the meninges, the spleen and the intact brain parenchyma, and flow cytometry shows that most γδ T cells (more than 98%) are positive for Ki67, indicating they are in the proliferative state. Thirdly, the components of the parenchymal γδ T cells 5 dpi of CMBs are decoded by Raman spectra analysis, showing they are in highly active state and enriched in substances for energy metabolism and nucleic acid synthesis. These results suggest that recruited γδ T cells in the brain parenchyma are further stimulated by the microenvironment to proceed proliferation and give birth to numerous new γδ T cells.

Neuroinflammation is the important pathological characteristics of brain damage, and the inflammatory response including the invasion of blood-borne leukocytes, the migration and proliferation of brain-resident microglia, and the activation of astrocytes would persist for weeks at the trauma site (39, 40). Our study indicates that CMBs-induced γδ T cells alleviate the local neuroinflammation in the brain parenchyma. RNAseq analysis of cortical samples shows the increase of the inflammatory signaling pathways from γδ T cell deficient mice compared to WT mice. Glial response is the indicator of neuroinflammation, and the CMBs induce the proliferation of astrocytes and microglia in the cortex. The cell density of reactive astrocytes and microglia is significantly enhanced in γδ T cell deficient mice compared to WT mice. Active microglia are the main source of macrophages in the injured brain, which are classified into proinflammatory M1 and anti-inflammatory M2 phenotypes. Immunostaining against iNOS and Arg1 (makers for M1 and M2 respectively) indicates that γδ T cell deficiency results in an increase of M1 macrophages and a decrease of M2 macrophages in the injured cortex after CMBs. All these results support that the recruited and proliferated γδ T cells in the brain parenchyma alleviate local neuroinflammation at the early stage of CMBs.

γδ T cells play an important role in regulating neuroinflammation through two main effectors of IFN-γ and IL-17A (15, 18). However, the effect of γδ T cells and their effectors on inflammation regulation and neurological outcomes remain controversial (38). Both beneficial and detrimental function of γδ T cells-derived IFN-γ in neuroinflammation are reported (41–43). It is reported that Vγ4 γδ T cells-derived IL-17A is involved in evoking the inflammatory cascade in the ischemic stroke (44). In the CNS injury models, the role of IL-17A is reported detrimental, which expression is induced by active microglia/macrophages-derived IL-23 at the secondary injury stage (19, 45), whereas IL-17A is also reported to benefit the survival and differentiation of neural precursor cells, and functional recovery of stroke at the delayed phases (46). The effects of γδ T cells may be dependent on the injury severity and the immune microenvironment. In the minor injury, our study shows that IL-17A^+^ γδ T cells are the main cell type in the brain parenchyma after CMBs and they play a beneficial role. After CMBs, γδ T cells in the brain parenchyma are active and maintain the ability of lipid fragment assimilation characterized by high expression of CD44 and CD36, which are the activation marker and lipid transport receptor respectively (34, 35).

CMBs result in the penetration of different cytokines into the brain parenchyma and are the early sign of the brain damage. With two-photon *in vivo* imaging, neuronal degeneration, manifested as degenerating dendritic bubbles, emerged in large numbers in the peri-CMBs region at 4 dpi, rare at the earlier timepoints. This should be due to CMBs-induced secondary damage to neurons. However, the onset of CMBs-induced neuron degeneration appears earlier (at 1 dpi) in γδ T cells deficient mice compared to WT mice. This further confirms that CMBs-induced γδ T cells in the parenchyma have a beneficial role of neuroprotection.

**Figure 7:**
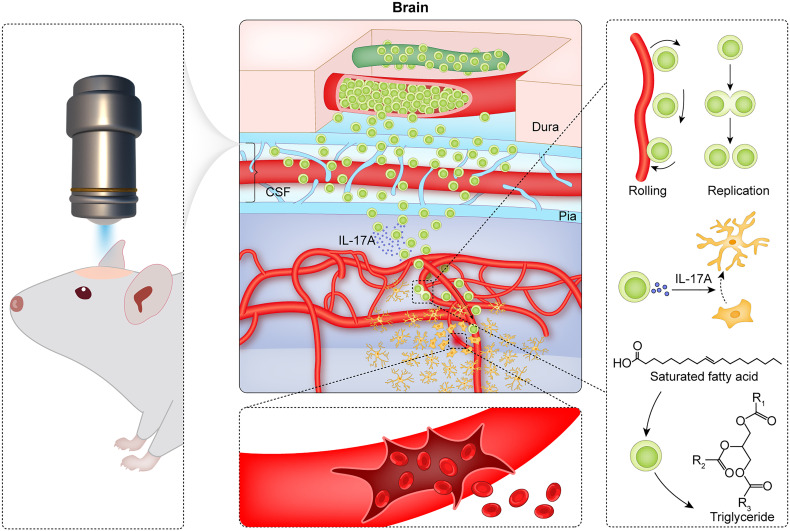
Summary of CMBs-induced γδ T cells in the brain parenchyma. Using a two-photon microscopy, a spot injury of the microvessel is made to mimic a model of CMBs, and the dynamic behaviors of γδ T cells are monitored. After CMBs, γδ T cells are recruited to the peri-CMBs brain parenchyma from the dural blood vessels and proliferate locally with an elevated lipid metabolism. γδ T cells secret IL-17A and play the roles of anti-neuroinflammation and neuroprotection at early stage of CMBs.

In conclusion, CMBs induce the accumulation of γδ T cells in the brain parenchyma where γδ T cells proliferate locally, absorb lipid, secrete factors, and play the roles of anti-neuroinflammation and neuroprotection (summarized in **Figure 7**). Modulation of γδ T cells is a potential strategy for preventing the early brain damage after CMBs.

## Data availability statement

The original contributions presented in the study are included in the article/**Supplementary Material**. Further inquiries can be directed to the corresponding author/s.

## Ethics statement

The animal study was reviewed and approved by Animal Ethics Committee of Jinan University. Written informed consent was obtained from the owners for the participation of their animals in this study.

## Author contributions

ZY, LBZ, LYZ and XL supervised the project; XS and SY performed the experiments, analyzed the data and prepared the draft; YL and QT assisted with mouse breeding and cellular experiments; ZX and SL helped with establishing mouse models; LBZ revised the manuscript; all authors read and approved the final manuscript. 

## References

[1] Wang B Chang M Zhang R Wo J Wu B Zhang H. Spinal cord injury target-immunotherapy with tnf-Α autoregulated and feedback-controlled human umbilical cord mesenchymal stem cell derived exosomes remodelled by Crispr/Cas9 plasmid. Materials Sci Engineering: C (2021) 133:112624. doi: 10.1016/j.msec.2021.112624 35525736

[2] Lo P Crouzet C Vasilevko V Choi B. Visualization of microbleeds with optical histology in mouse model of cerebral amyloid angiopathy. Microvascular Res (2016) 105:109–13. doi: 10.1016/j.mvr.2016.02.002 PMC4814270 26876114

[3] Merlini M Rafalski VA Rios Coronado PE Gill TM Ellisman M Muthukumar G. Fibrinogen induces microglia-mediated spine elimination and cognitive impairment in an alzheimer’s disease model. Neuron (2019) 101(6):1099–108.e6. doi: 10.1016/j.neuron.2019.01.014 30737131 PMC6602536

[4] Rosidi NL Zhou J Pattanaik S Wang P Jin W Brophy M. Cortical microhemorrhages cause local inflammation but do not trigger widespread dendrite degeneration. PloS One (2011) 6(10):e26612. doi: 10.1371/journal.pone.0026612 22028924 PMC3197572

[5] He X-F Lan Y Zhang Q Liu D-X Wang Q Liang F-Y. Deferoxamine inhibits microglial activation, attenuates blood-brain barrier disruption, rescues dendritic damage, and improves spatial memory in a mouse model of microhemorrhages. J Neurochemistry (2016) 138(3):436–47. doi: 10.1111/jnc.13657 27167158

[6] Pantoni L. Cerebral small vessel disease: From pathogenesis and clinical characteristics to therapeutic challenges. Lancet Neurol (2010) 9(7):689–701. doi: 10.1016/s1474-4422(10)70104-6 20610345

[7] Rustenhoven J Drieu A Mamuladze T De Lima KA Dykstra T Wall M. Functional characterization of the dural sinuses as a neuroimmune interface. Cell (2021) 184(4):1000–16.e27. doi: 10.1016/j.cell.2020.12.040 33508229 PMC8487654

[8] Alves de Lima K Rustenhoven J Kipnis J. Meningeal immunity and its function in maintenance of the central nervous system in health and disease. Annu Rev Immunol (2020) 38:597–620. doi: 10.1146/annurev-immunol-102319-103410 32340575

[9] Shi L Sun Z Su W Xu F Xie D Zhang Q. Treg cell-derived osteopontin promotes microglia-mediated white matter repair after ischemic stroke. Immunity (2021) 54(7):1527–42.e8. doi: 10.1016/j.immuni.2021.04.022 34015256 PMC8282725

[10] Liesz A Suri-Payer E Veltkamp C Doerr H Sommer C Rivest S. Regulatory T cells are key cerebroprotective immunomodulators in acute experimental stroke. Nat Med (2009) 15(2):192–9. doi: 10.1038/nm.1927 19169263

[11] Zhang D Ren J Luo Y He Q Zhao R Chang J. T Cell response in ischemic stroke: From mechanisms to translational insights. Front Immunol (2021) 12:707972. doi: 10.3389/fimmu.2021.707972 34335623 PMC8320432

[12] Ahn SJ Anrather J Nishimura N Schaffer CB. Diverse inflammatory response after cerebral microbleeds includes coordinated microglial migration and proliferation. Stroke (2018) 49(7):1719–26. doi: 10.1161/STROKEAHA.117.020461 PMC6019563 29844029

[13] Liu L Dodd S Hunt RD Pothayee N Atanasijevic T Bouraoud N. Early detection of cerebrovascular pathology and protective antiviral immunity by mri. eLife (2022) 11. doi: 10.7554/elife.74462 PMC9106335 35510986

[14] Shiromizu CM Jancic CC. Gammadelta T lymphocytes: An effector cell in autoimmunity and infection. Front Immunol (2018) 9:2389. doi: 10.3389/fimmu.2018.02389 30386339 PMC6198062

[15] Sun G Yang S Cao G Wang Q Hao J Wen Q. Γδ T cells provide the early source of ifn-Γ to aggravate lesions in spinal cord injury. J Exp Med (2018) 215(2):521–35. doi: 10.1084/jem.20170686 PMC5789408 29282251

[16] Benakis C Brea D Caballero S Faraco G Moore J Murphy M. Commensal microbiota affects ischemic stroke outcome by regulating intestinal Γδ T cells. Nat Med (2016) 22(5):516–23. doi: 10.1038/nm.4068 PMC4860105 27019327

[17] Arunachalam P Ludewig P Melich P Arumugam TV Gerloff C Prinz I. Ccr6 (Cc chemokine receptor 6) is essential for the migration of detrimental natural Interleukin-17–producing Γδ T cells in stroke. Stroke (2017) 48(7):1957–65. doi: 10.1161/strokeaha.117.016753 28611085

[18] Zhong Q Zhou K Liang QL Lin S Wang YC Xiong XY. Interleukin-23 secreted by activated macrophages drives Γδt cell production of interleukin-17 to aggravate secondary injury after intracerebral hemorrhage. J Am Heart Assoc (2016) 5(10):e004340. doi: 10.1161/jaha.116.004340 27729335 PMC5121525

[19] Shichita T Sugiyama Y Ooboshi H Sugimori H Nakagawa R Takada I. Pivotal role of cerebral Interleukin-17–producing Γδt cells in the delayed phase of ischemic brain injury. Nat Med (2009) 15(8):946–50. doi: 10.1038/nm.1999 19648929

[20] Li Y Zhang Y Zeng X. Gammadelta T cells participating in nervous systems: A story of Jekyll and Hyde. Front Immunol (2021) 12:656097. doi: 10.3389/fimmu.2021.656097 33868300 PMC8044362

[21] Nishimura N Schaffer CB Friedman B Tsai PS Lyden PD Kleinfeld D. Targeted insult to subsurface cortical blood vessels using ultrashort laser pulses: Three models of stroke. Nat Methods (2006) 3(2):99–108. doi: 10.1038/nmeth844 16432519

[22] Ahn JH Cho H Kim J-H Kim SH Ham J-S Park I. Meningeal lymphatic vessels at the skull base drain cerebrospinal fluid. Nature (2019) 572(7767):62–6. doi: 10.1038/s41586-019-1419-5 31341278

[23] Louveau A Herz J Alme MN Salvador AF Dong MQ Viar KE. Cns lymphatic drainage and neuroinflammation are regulated by meningeal lymphatic vasculature. Nat Neurosci (2018) 21(10):1380–91. doi: 10.1038/s41593-018-0227-9 PMC6214619 30224810

[24] Alves De Lima K Rustenhoven J Da Mesquita S Wall M Salvador AF Smirnov I. Meningeal Γδ T cells regulate anxiety-like behavior *Via* il-17a signaling in neurons. Nat Immunol (2020) 21(11):1421–9. doi: 10.1038/s41590-020-0776-4 PMC8496952 32929273

[25] Mannie MD McConnell TJ Xie C Li Y-Q. Activation-dependent phases of T cells distinguished by use of optical tweezers and near infrared raman spectroscopy. J Immunol Methods (2005) 297(1-2):53–60. doi: 10.1016/j.jim.2004.11.020 15777930

[26] Movasaghi Z Rehman S Rehman IU. Raman spectroscopy of biological tissues. Appl Spectrosc Rev (2007) 42(5):493–541. doi: 10.1080/05704920701551530

[27] Ichimura T Chiu L-D Fujita K Machiyama H Yamaguchi T Watanabe TM. Non-label immune cell state prediction using raman spectroscopy. Sci Rep (2016) 6(1):37562. doi: 10.1038/srep37562 27876845 PMC5120326

[28] Czamara K Majzner K Pacia MZ Kochan K Kaczor A Baranska M. Raman spectroscopy of lipids: A review. J Raman Spectrosc (2015) 46(1):4–20. doi: 10.1002/jrs.4607

[29] Veloso A Fernández R Astigarraga E Barreda-Gómez G Manuel I Giralt MT. Distribution of lipids in human brain. Analytical Bioanalytical Chem (2011) 401(1):89–101. doi: 10.1007/s00216-011-4882-x 21437774

[30] Wu Y Wu W Wong WM Ward E Thrasher AJ Goldblatt D. Human gamma delta T cells: A lymphoid lineage cell capable of professional phagocytosis. J Immunol (2009) 183(9):5622–9. doi: 10.4049/jimmunol.0901772 19843947

[31] Holderness J Hedges JF Ramstead A Jutila MA. Comparative biology of gammadelta T cell function in humans, mice, and domestic animals. Annu Rev Anim Biosci (2013) 1:99–124. doi: 10.1146/annurev-animal-031412-103639 25387013

[32] Barisa M Kramer AM Majani Y Moulding D Saraiva L Bajaj-Elliott M. E. coli promotes human Vγ9vδ2 T cell transition from cytokine-producing bactericidal effectors to professional phagocytic killers in a tcr-dependent manner. Sci Rep (2017) 7(1):2805. doi: 10.1038/s41598-017-02886-8 28584241 PMC5459831

[33] Howie D Ten Bokum A Necula AS Cobbold SP Waldmann H. The role of lipid metabolism in T lymphocyte differentiation and survival. Front Immunol (2017) 8:1949. doi: 10.3389/fimmu.2017.01949 29375572 PMC5770376

[34] Xu S Chaudhary O Rodríguez-Morales P Sun X Chen D Zappasodi R. Uptake of oxidized lipids by the scavenger receptor Cd36 promotes lipid peroxidation and dysfunction in Cd8+ T cells in tumors. Immunity (2021) 54(7):1561–77.e7. doi: 10.1016/j.immuni.2021.05.003 34102100 PMC9273026

[35] DeGrendele HC Kosfiszer M Estess P Siegelman MH. Cd44 activation and associated primary adhesion is inducible *Via* T cell receptor stimulation. J Immunol (1997) 159(6):2549–53. 9300670

[36] Tavares GA Louveau A. Meningeal lymphatics: An immune gateway for the central nervous system. Cells (2021) 10(12):3385. doi: 10.3390/cells10123385 34943894 PMC8699870

[37] Schläger C Körner H Krueger M Vidoli S Haberl M Mielke D. Effector T-cell trafficking between the leptomeninges and the cerebrospinal fluid. Nature (2016) 530(7590):349–53. doi: 10.1038/nature16939 26863192

[38] Wang L Yao C Chen J Ge Y Wang C Wang Y. Γδ T cell in cerebral ischemic stroke: Characteristic, immunity-inflammatory role, and therapy. Front Neurol (2022) 13:842212. doi: 10.3389/fneur.2022.842212 35432162 PMC9008352

[39] Shtaya A Bridges LR Esiri MM Lam-Wong J Nicoll JAR Boche D. Rapid neuroinflammatory changes in human acute intracerebral hemorrhage. Ann Clin Trans Neurol (2019) 6(8):1465–79. doi: 10.1002/acn3.50842 PMC6689697 31402627

[40] Dasari R Bonsack F Sukumari-Ramesh S. Brain injury and repair after intracerebral hemorrhage: The role of microglia and brain-infiltrating macrophages. Neurochem Int (2021) 142:104923. doi: 10.1016/j.neuint.2020.104923 33248206 PMC7818651

[41] Zhang J He H Qiao Y Zhou T He H Yi S. Priming of microglia with ifn-gamma impairs adult hippocampal neurogenesis and leads to depression-like behaviors and cognitive defects. Glia (2020) 68(12):2674–92. doi: 10.1002/glia.23878 32652855

[42] Kunis G Baruch K Rosenzweig N Kertser A Miller O Berkutzki T. Ifn-Gamma-Dependent activation of the brain's choroid plexus for cns immune surveillance and repair. Brain (2013) 136(Pt 11):3427–40. doi: 10.1093/brain/awt259 24088808

[43] Roselli F Chandrasekar A Morganti-Kossmann MC. Interferons in traumatic brain and spinal cord injury: Current evidence for translational application. Front Neurol (2018) 9:458. doi: 10.3389/fneur.2018.00458 29971040 PMC6018073

[44] Lu L Wang Y Zhou L Li Y Zhang X Hu X. Vγ4 T cell-derived il-17a is essential for amplification of inflammatory cascades in ischemic brain tissue after stroke. Int Immunopharmacol (2021) 96:107678. doi: 10.1016/j.intimp.2021.107678 34162129

[45] Yang Z Liu Q Shi H Jiang X Wang S Lu Y. Interleukin 17a exacerbates er-Stress-Mediated inflammation of macrophages following ich. Mol Immunol (2018) 101:38–45. doi: 10.1016/j.molimm.2018.05.020 29859495

[46] Lin Y Zhang JC Yao CY Wu Y Abdelgawad AF Yao SL. Critical role of astrocytic interleukin-17 a in post-stroke survival and neuronal differentiation of neural precursor cells in adult mice. Cell Death Dis (2016) 7(6):e2273. doi: 10.1038/cddis.2015.284 27336717 PMC5143370

