# Supplementary material for: γδ T cells recruitment and local proliferation in brain parenchyma benefit anti-neuroinflammation after cerebral microbleeds
Source: Front Immunol. 2023 Mar 29;14:1139601. doi: 10.3389/fimmu.2023.1139601 (PMC10090560; doi:10.3389/fimmu.2023.1139601)
Supplement: Supplementary file 1 [file DataSheet_1.docx]

**Supplementary Materials**

**γδ T cells recruitment and local proliferation in brain parenchyma benefit anti-neuroinflammation after cerebral microbleeds**

**
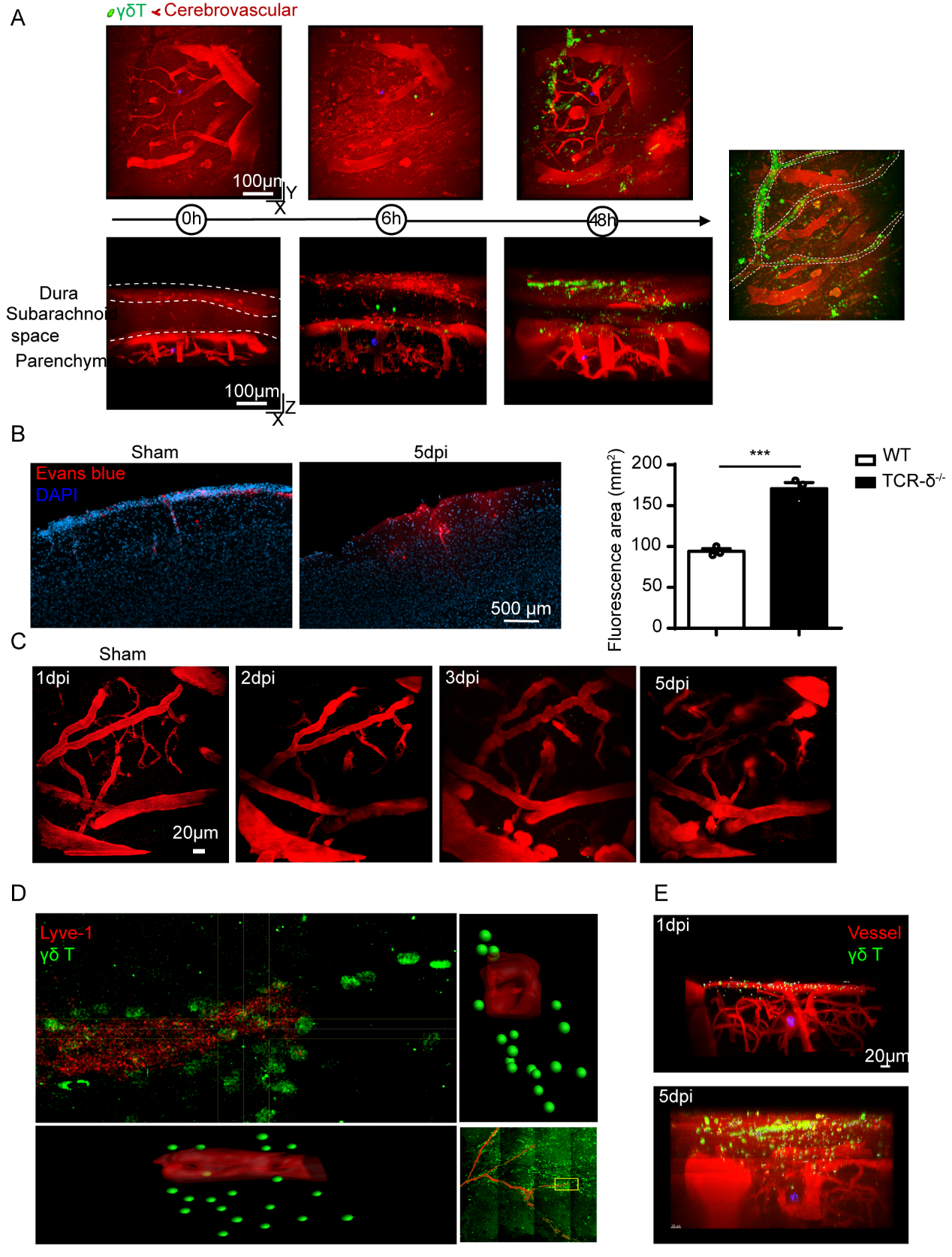
**

**Supplementary Fig. 1 Visualizing the** **permeability of dural blood vessels and the distribution of γδ T cells.**

(A) The distribution of γδ T cells (green) is tracked by *in vivo* imaging at 0, 6 and 48 h after CMBs from the vertical (3 upper panels) and lateral (3 lower panels) view. Reconstructed image (far right) shows numerous γδ T cells inside the dural blood vessels (outline by dotted lines) and some out of the vessel lumen. Blood vessels are labelled by rhodamine in red. (B) Evans blue staining (red) shows the increased permeability of dural blood vessels in the cortex after CMBs compared with that from the sham animal, and statistical quantification of fluorescence-coverage is significant different (*** *P*<0.001, Student’s *t*-test, n=3). (C) In the sham animal (*TCR-δ^EGFP^* mouse with a cranial window open only), two-photon images show no γδ T cells (green) at different days post injury (dpi) and the blood vessels are readily identified by rhodamine (red). (D) In the meninges of *TCR-δ^EGFP^* mice at 5 dpi of CMBs, the relative location of meningeal lymphatic vessels (red) and γδ T cells (green) is identified by anti-Lyve-1 immunostaining and EGFP signal. (E) Extended focus view of two-photon image shows the moving of γδ T cells (green) out of the dural blood vessels and towards the injured site (blue spot) at 1 and 5 dpi. The blood vessels are labelled by rhodamine in red.

**
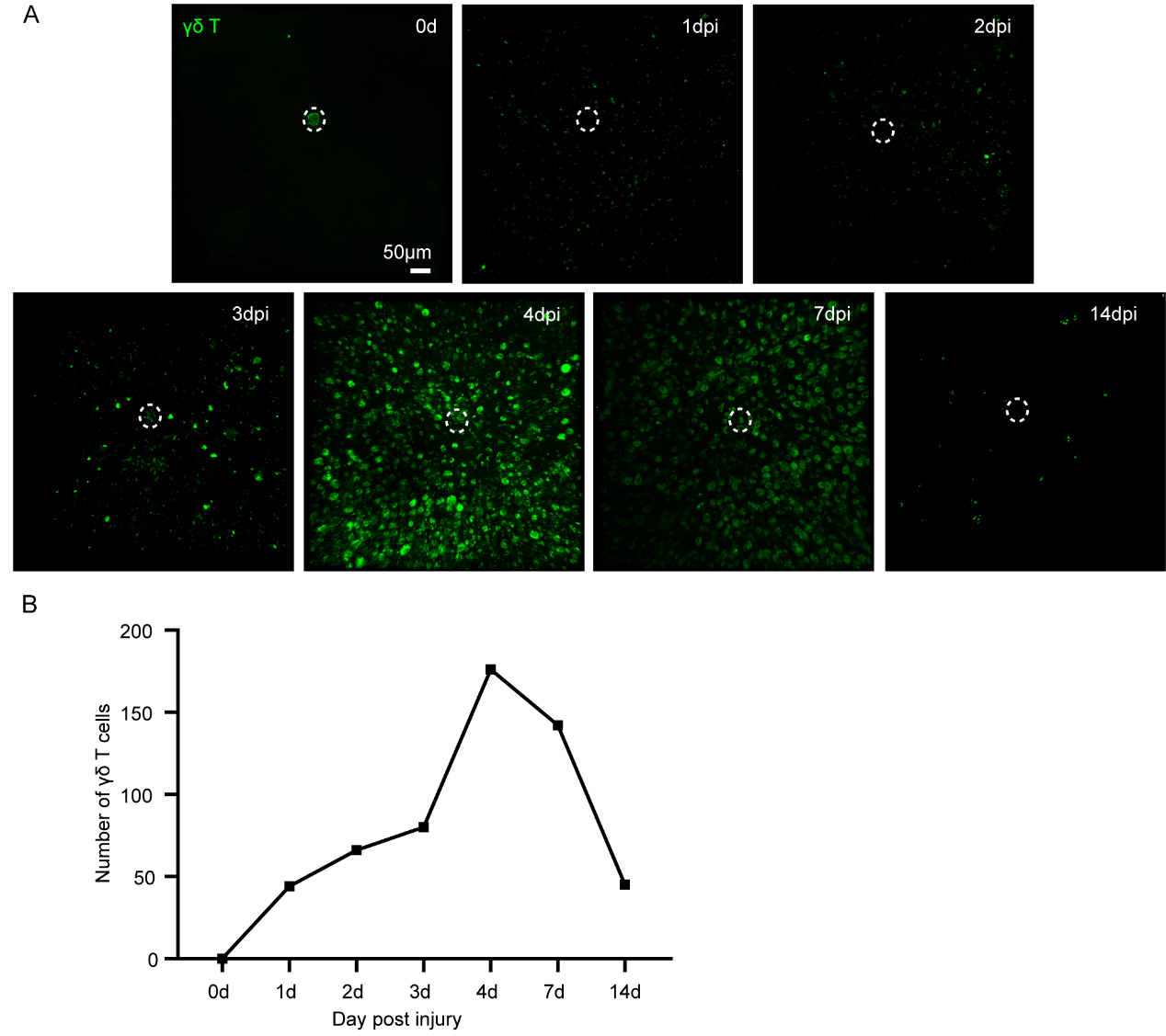
**

**Supplementary Fig. 2 Vein delivery of cultured γδ T cells invade the peri-CMBs brain parenchyma.** (A) Cultured γδ T cells from *TCR-δ^EGFP^* mice are transplanted to *TCR-δ^-/-^* mice through tail vein injection, and the distribution of γδ T cells (green) surrounding the CMBs (dotted circles) are visualized at different days post injury (dpi) by two-photon imaging. (B) The graph shows the numbers of γδ T cells in the brain at different timepoints after CMBs. N=3 animals in each group.


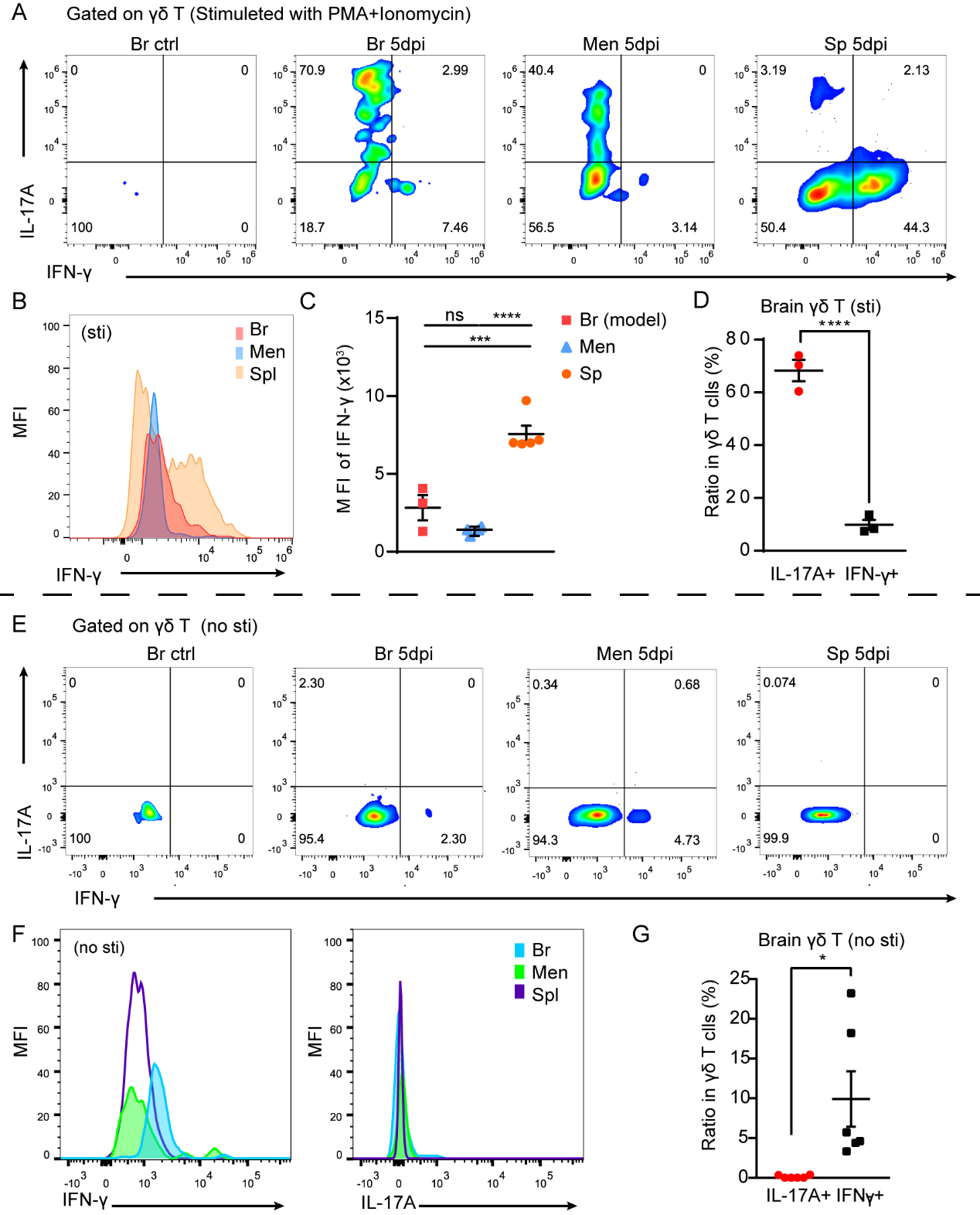


**Supplementary Fig. 3 γδ T cells in peri-CMBs brain parenchyma are characterized by the expression of IL-17A with intracellular stimulation.**

(A-D) Flow cytometry identifies high expression of IL-17A in γδ T cells from peri-CMBs brain parenchymal samples (Br) compared to meninges (Men), intact brain (Ctrl), and spleen (Spl) at 5 dpi with intracellular stimulation (sti). One sample from at least 3 mice and n≥3 samples in each group; *** *P*<0.001, **** *P*<0.0001; One-way ANOVA with Tukey’s multiple comparison, Student’s *t*-test for two-group comparison. (E-G) Flow cytometry identifies low expression of IL-17A and high expression of IFN-γ in γδ T cells from peri-CMBs brain parenchymal samples (Br) and meninges (Men) compared to intact brain (Ctrl), and spleen (Spl) at 5 dpi, intrinsically (no stimulation: no sti). The representative picture of flowcytometry(E) and MFI (F) of IL-17A expression in γδ T cells from different tissues. (G) Statistic shows that the percentage of IFN-γ^+^ γδ T cells is intrinsically higher than that of IL-17A^+^ γδ T cells in injured brain parenchyma-derived γδ T cell without stimulation. * *P*<0.05; Student’s *t*-test; one sample from 4-6 mice and 3 samples in each group.
